# Supplementary figures and images for: Beyond the MEP Pathway: A novel kinase required for prenol utilization by malaria parasites
Source: PLoS Pathog. 2024 Jan 26;20(1):e1011557. doi: 10.1371/journal.ppat.1011557 (PMC10849223; doi:10.1371/journal.ppat.1011557)

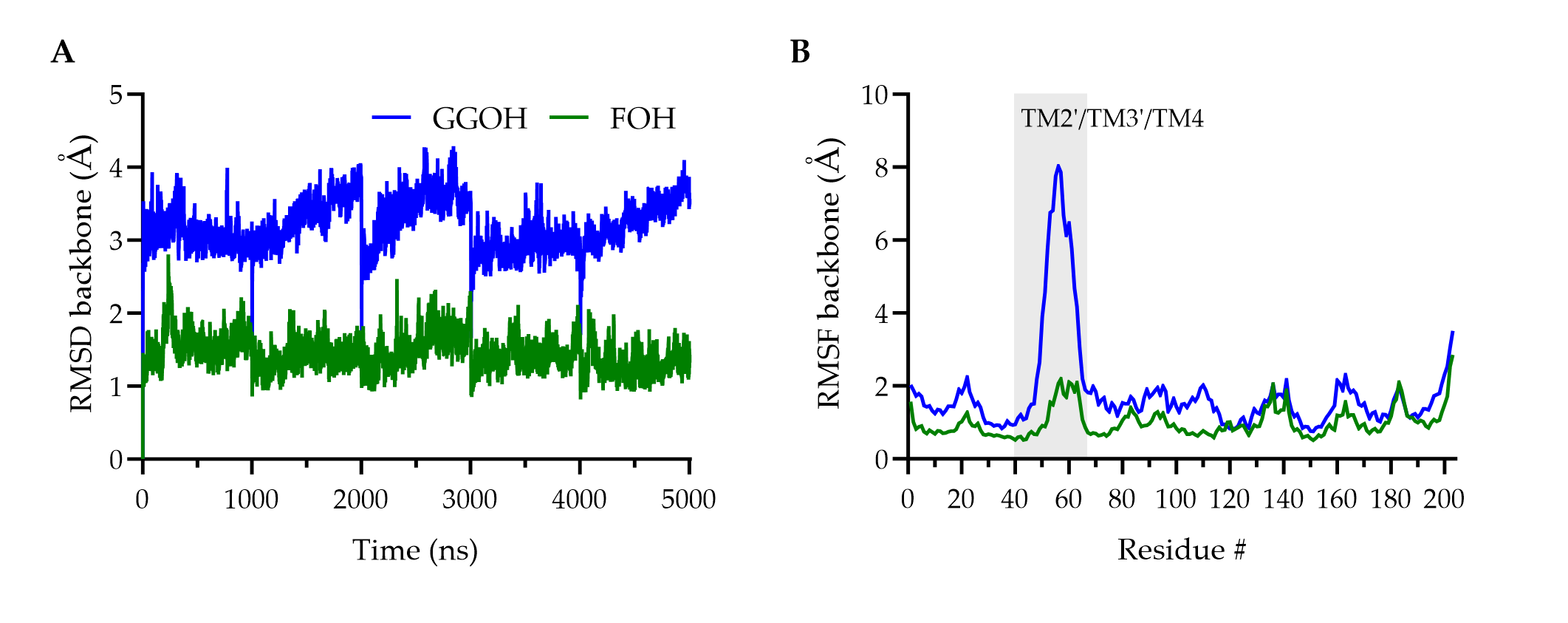

Supplement: S1 Fig — A) Root mean square deviation (RMSD) values of the protein backbone were used to monitor simulation equilibration and protein changes along the trajectory time (merged 5x1 μs). B) Root mean square fluctuation (RMSF) by residues, calculated using the initial MD frame as a reference and compared between ligand-bound, highlighting the TM2’-TM4 region (the intracellular portion) which displays a unique unfolding in the GGOH simulations. (TIF) [file ppat.1011557.s001.tif]

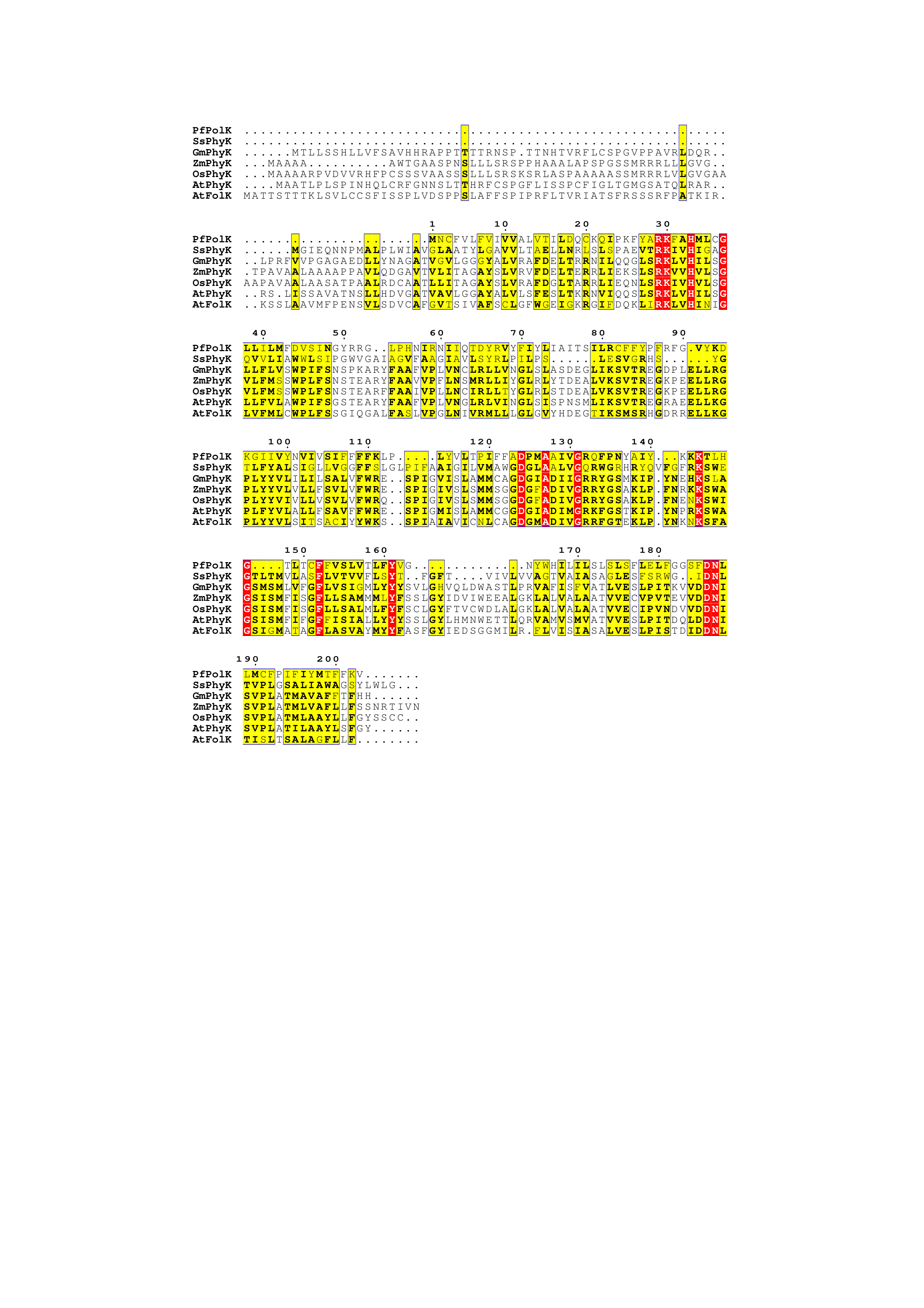

Supplement: S2 Fig — ClustalW Multiple alignment of PolK candidate amino acid sequence, predicted in P. falciparum NF54 strain against sequences with prenol kinase prediction in UniProt database. PolK: prenol kinase, PhyK: phytol kinase, FolK: farnesol kinase. PfPolK (P. falciparum NF54, D0VEH1), SsPhyK (Synechocystis sp., P74653), GmPhyK (Glycine max, Q2N2K1), ZmPhyK (Zea mays, Q2N2K4), OsPhyK (Oryza sativa, Q7XR51), AtPhyK (Arabidopsis thaliana, Q9LZ76), AtFolK (Arabidopsis thaliana, Q67ZM7). Yellow indicates similarity and red indicates identity. (TIF) [file ppat.1011557.s002.tif]

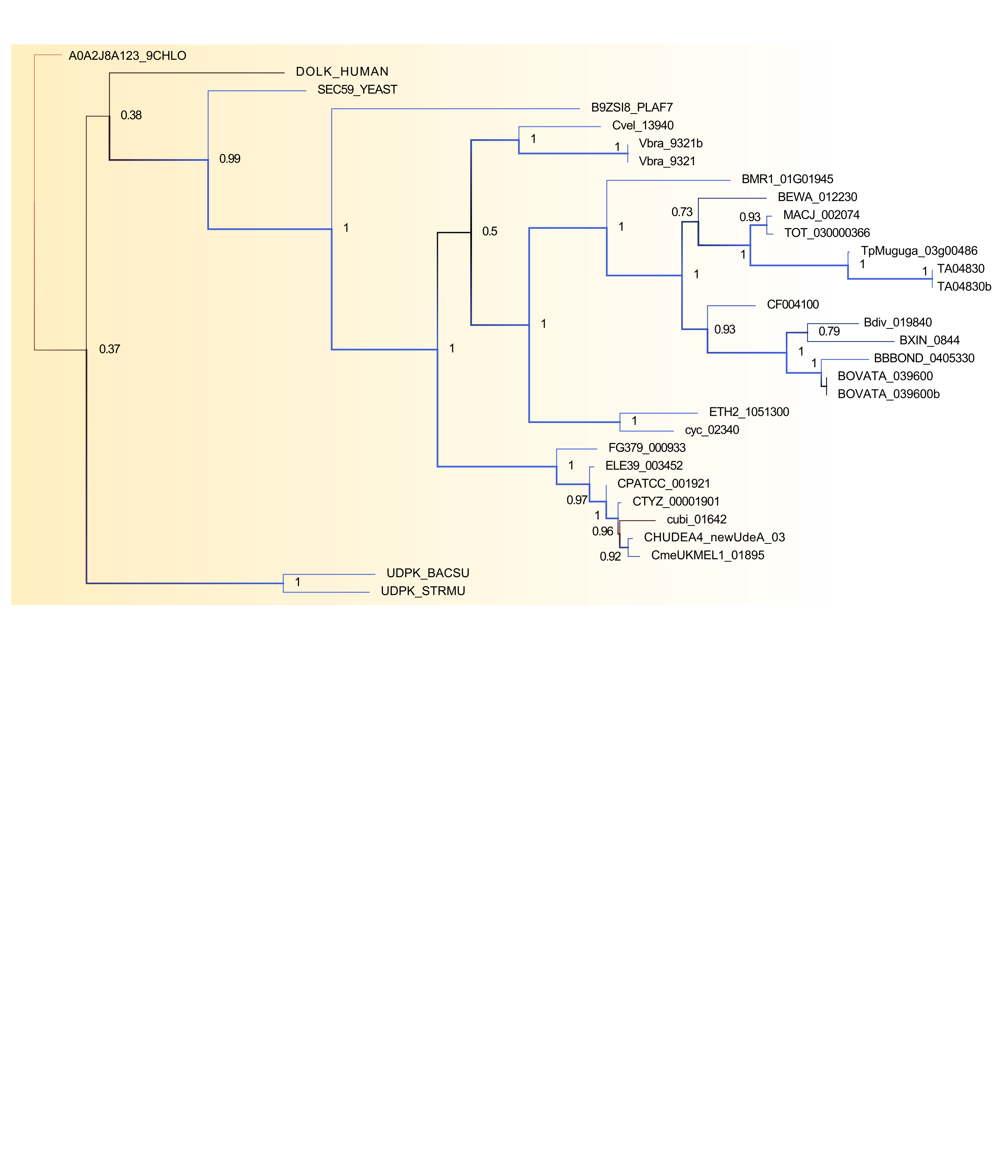

Supplement: S3 Fig — Inset of the overall phylogenetic dendrogram of potential prenol kinases generated using maximum likelihood method (see Methods). Branch support values (Bayes posterior probability) are displayed as numbers for the most relevant clade separation, as well as colours (from the highest scores, in blue, to the lowest values, in red) and thickness of the branches. Organisms and genes from Opisthokont group and some extra outliers are highlighted. (TIF) [file ppat.1011557.s003.tif]

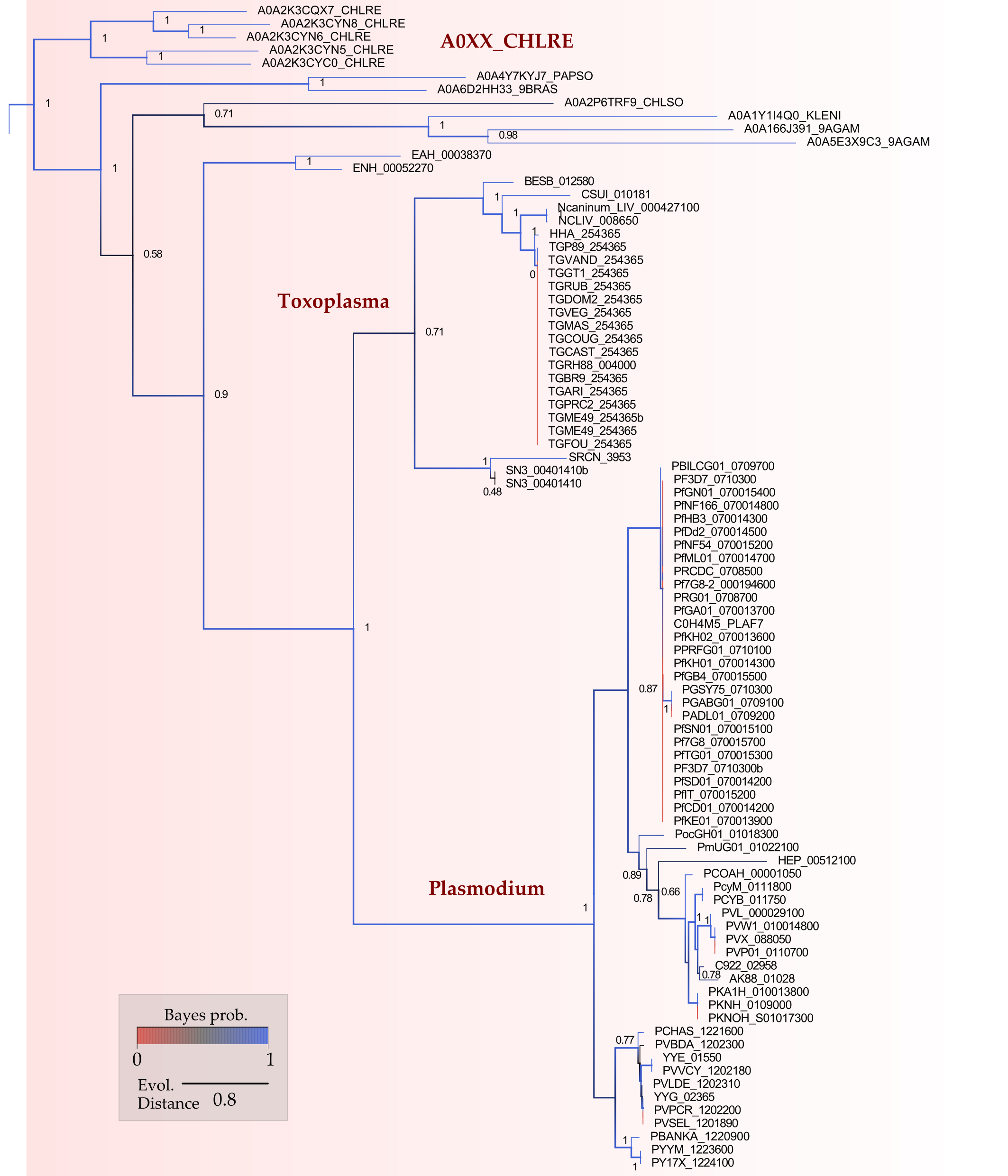

Supplement: S4 Fig — Inset of the overall phylogenetic dendrogram of potential prenol kinases generated using maximum likelihood method (see Methods). Branch support values (Bayes posterior probability) are displayed as numbers for the most relevant clade separation, as well as colours (from the highest scores, in blue, to the lowest values, in red) and thickness of the branches. Apicomplexa clades and the C. reinhardtii (A0XX_CHLRE, where A0XX is a generic label for all the C. reinhardtii’s taxa) are highlighted. (TIF) [file ppat.1011557.s004.tif]

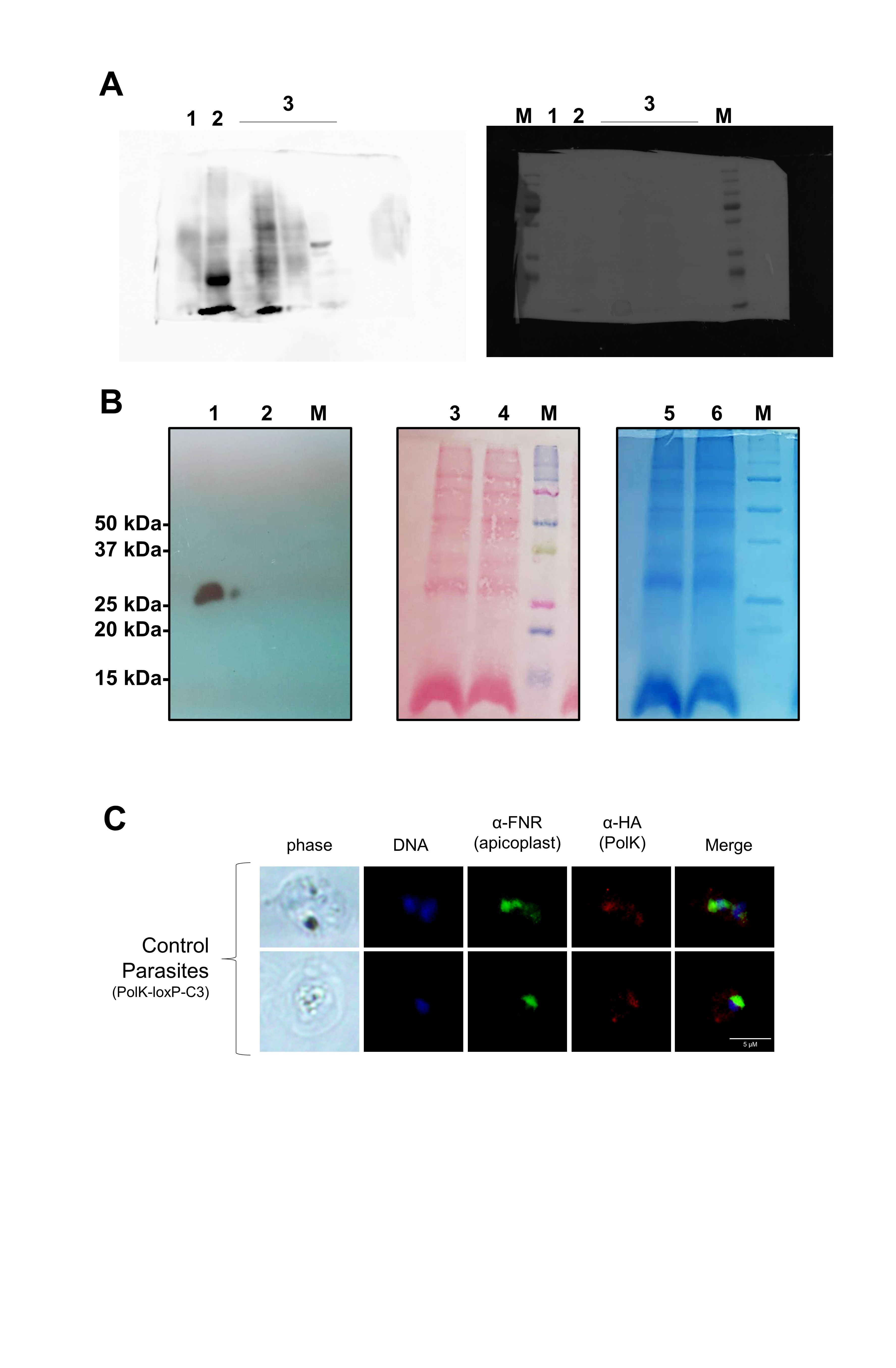

Supplement: S5 Fig — (A) Photographs of a Western blot of transgenic parasites (left) and the respective PVDF membrane (right) with the protein ladder (M). The Western blot was performed to analyze the HA-tagged PfPolK in parasites where PfPolK was excised (Lane 1, parasites exposed to rapamycin) or preserved (Lane 2, parasites exposed to DMSO). The remaining lanes (group of lanes 3 and onwards) correspond to experiments not related to this article. (B) This panel displays photographs of a Western blot of transgenic parasites (left), the respective Ponceau S staining of the PVDF membrane post-transfer (center), and the protein bands visualized on the Coomassie-stained gel (right). The Precision Plus Protein Kaleidoscop Prestained Protein Standards (BioRad, #1610375) was used to indicate the molecular mass. The Western blot was conducted to analyze the HA-tagged PfPolK in parasites where PfPolK was excised (Lanes 1, 3, and 5, parasites exposed to rapamycin) or preserved (Lanes 2, 4, and 6, parasites exposed to DMSO). (C) Immunofluorescence analysis was conducted on HA-tagged PfPolK parasites. In this analysis, HA-tagged PfPolK is marked in red using α-HA (PolK), the apicoplast is indicated in green using α-FNR (apicoplast), and the nucleus maked in blue using Hoechst 33342 (DNA). The analysis was performed on parasites at the trophozoite stage. (TIF) [file ppat.1011557.s005.tif]

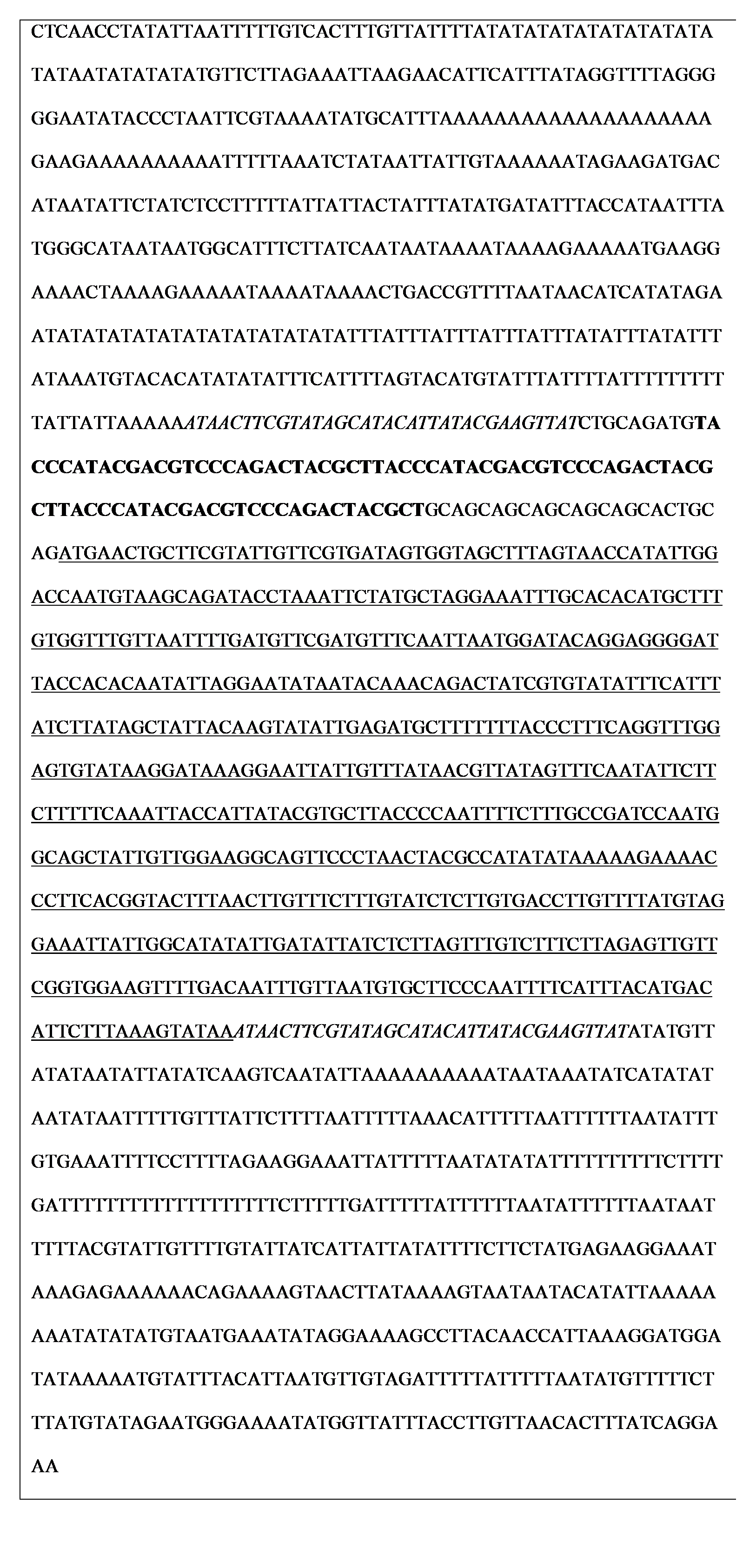

Supplement: S6 Fig — The sequence displays the cassete composed by the recodonized version of PfPolk (underlined) and a 3x-HA sequence in the 5’ end (bold), all flanked by two loxP sites (italics) and the two homology regions upstream and downstream of the cassete (regular). This sequence is part of the transfection plasmid sequence used in this research. (TIF) [file ppat.1011557.s006.tif]

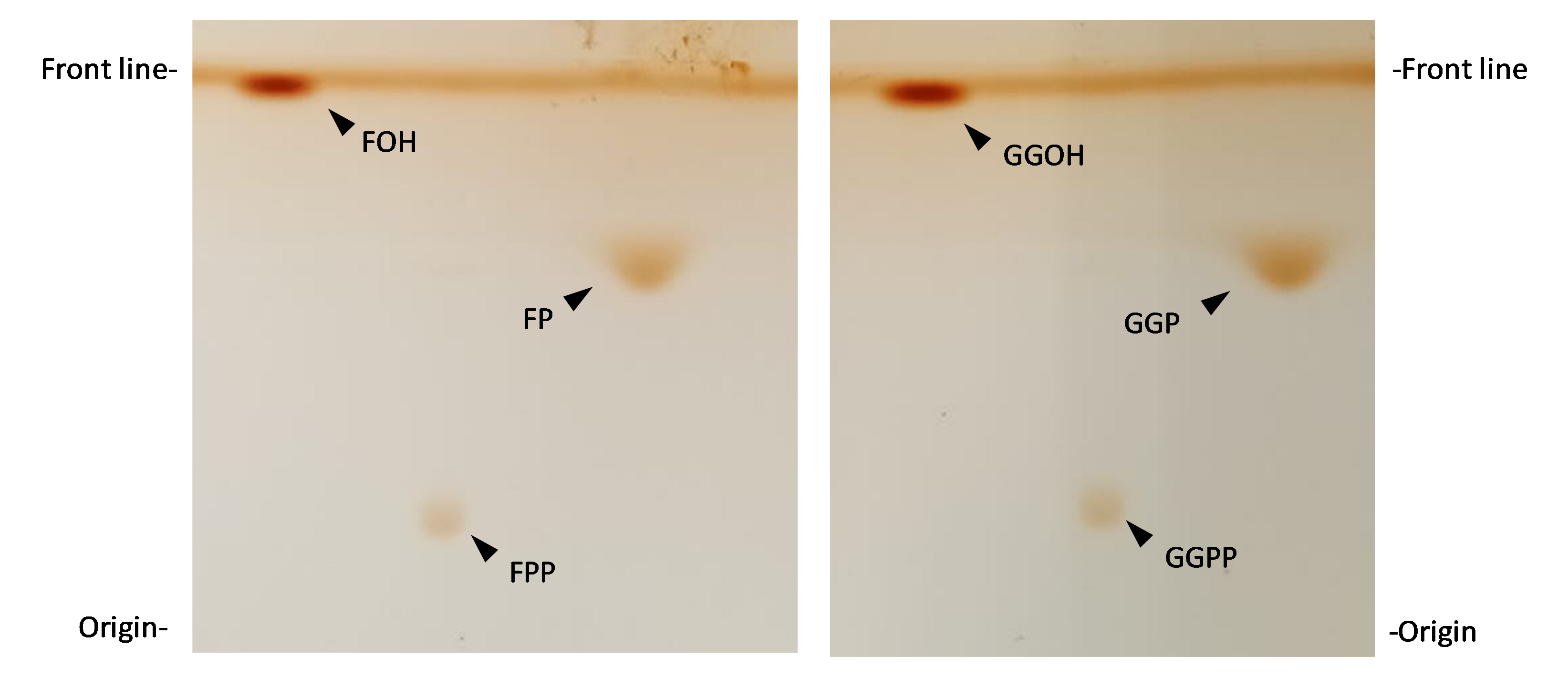

Supplement: S7 Fig — The images show the retention of different standards in TLC plates, as indicated. Standards were visualized using iodine vapor and UV light. (TIF) [file ppat.1011557.s007.tif]
