# Supplementary material for: Beyond the MEP Pathway: A novel kinase required for prenol utilization by malaria parasites
Source: PLoS Pathog. 2024 Jan 26;20(1):e1011557. doi: 10.1371/journal.ppat.1011557 (PMC10849223; doi:10.1371/journal.ppat.1011557)
Supplement: Attachment — Submitted filename: Author responses 11.docx [file ppat.1011557.s008.docx]

**Author´s responses to reviewers**

**Authors:** Thank you for providing the reviews for our manuscript. We are grateful for the thorough and helpful way the paper has been reviewed. By comprehensively attending the reviewers’ critiques and comments, we believe the paper has been much strengthened. We have addressed all the suggested points, and to the best of our capabilities, we have carried out key recommended experiments. We have also identified a mistake -the use of ^14^C isoleucine instead of ^3^H isoleucine- that has been now corrected (see below). Our precise responses to the reviewers are laid out below, marked with a grey background. We hope that the revised manuscript will now be acceptable for PloS Pathogens and we look forward to hearing from you.

**Part I - Summary**

***Reviewer 1:*** *The manuscript by Crispim et al. describes the function of a conserved un-annotated Plasmodium gene as being a prenol kinase with the ability to phosphorylate farnesol (FOH) and geranylgeraniol (GGOH). This group has previously reported enzymatic activity in P. falciparum lysate that can produce farnesyl pyrophosphate (FPP) from FOH. This result raises a provocative possibility of a prenol salvage capacity in malaria parasites. Since isopentyl pyrophosphate (IPP), the precursor FPP and other polyprenyl pyrophosphates, is synthesized through methylerythritol phosphate (MEP) pathway, it has been investigated as an attractive target for antimalarial drugs such as fosmidomycin. A salvage bypass, as suggested by the authors, could pose a potential challenge for discovering MEP pathway inhibitors. Thus, the findings are provocative and of considerable significance. There are several minor and moderate concerns that authors need to address. I list them in order of their appearance in the manuscript.*

**Authors:** We deeply value the feedback provided by the reviewer. We concur that the primary focus of our article centers around the discovery of the PolK enzyme. As the reviewer pointed out, this discovery opens up the study of a prenol salvage mechanism in malaria parasites, which could be harnessed to enhance the effectiveness of specific antimalarial treatments. Please, note that we included new results supporting this hypothesis (Figure 7). It's important to emphasize that while this possibility remains unconfirmed, our paper lays the groundwork for future investigations in this direction. Currently, we firmly believe that it is crucial to disseminate the discovery of PolK so that the scientific community can delve into its potential role in *Plasmodium* or study PfPolk homologous genes in other organisms.

*1. Line 27 in Abstract: a fosmidomycin/clindamycin combination is not a “promising” treatment, as evidenced by references cited by the authors on lines 98-101. Authors should moderate this statement and also on lines 55 and 56.*

**Authors:** We appreciate the reviewer for pointing out the inconsistency. We have made the suggested change in line 29 to "A proposed treatment for malaria involves a combination of fosmidomycin and clindamycin." Additionally, we have removed the phrase as can be seen in lines 44-47, as we determined it to be unnecessary.

*2. In Figure 1, DNA gyrase inhibitors should be added along with ribosome inhibitors of apicoplast functions.*

**Authors:** We concur with the reviewer's suggestion, and as a result, we have incorporated DNA gyrase inhibitors into Figure 1. We have also mentioned these compounds in the main text and provided a reference (*Tan, S., Mudeppa, D. G., Kokkonda, S., White III, J., Patrapuvich, R., & Rathod, P. K. (2021). Properties of Plasmodium falciparum with a deleted apicoplast DNA gyrase. Antimicrobial agents and chemotherapy, 65(9), 10-1128.*). Now the text reads (lines 92-95) “Similarly, classic bacterial ribosome inhibitors (hereafter referred as ribosome inhibitors), such as azithromycin, doxycycline, or clindamycin, as well as DNA gyrase inhibitors (Tan et al., 2021) can indirectly inhibit isoprenoid biosynthesis by interfering with apicoplast biogenesis.” Finally, we also made other modifications in Figure 1, in order to demonstrate the IPP/DMAPP transport outside the Apicoplast.

*3. I did not see that the authors had included Chromera, dinoflagellates and ciliates in their phylogenetic analysis. These are likely to be more closely related to apicomplexans. Also, the statement that apicomplexans are derived from plants and C. reinhardtii (lines 350-353) is incorrect; apicomplexans are members of the Alveolata clade.*

**Authors:** We agree with the changes suggested and have generated a new phylogenetic tree (see corrected figure 2A). As to the specific statement, we have now modified it in the Discussion and removed the statement (lines 434-443) and changed the Results section 2.1.

*4. Figure 2D is repeated twice, the last panel should be “E” (figure 2 and line 218).*

**Authors:** We have now corrected this mistake, now in the line 225.

*5. Line 228: Was an epitope tag added to the gene? It might be worthwhile to state that the gene was codon optimized for yeast expression.*

**Authors:** We agree with the epitope-tagging approach suggested by the reviewer, but did not add a tag in order to maximize the chances of PfPolK active expression. We have now included this explanation, making also specific reference to the codon optimization, in the main text (lines 248-250) “To enhance the likelihood of actively expressing *Pf*PolK, no tags were added, and the *Pf*PolK gene sequence was optimized for recombinant expression.”. Additionally, we have included the optimized *Pf*PolK sequence in the supplemental data (Figure S5).

*6. Figure 3: What was the level of expression of PfPolK in the yeast? A western blot showing correct size protein expression without degradation should be included.*

**Authors:** The expression of the PolK enzyme presents challenges due to its multiple transmembrane domains, and the process of optimizing its enzymatic activity is costly. Given these substantial hurdles, we decided to enhance the likelihood of successfully expressing *Pf*PolK. In light of these considerations, we did not incorporate epitope tags for Western blot analysis and did not pursue the development of antibodies against *Pf*PolK, as we anticipated important challenges in achieving the purification of the enzyme. Remarkably, similar strategies have been embraced by several other authors who have explored prenol kinases in plant systems (PMID: 16361393; 21395888 ; 26452599).

*7. Figure 3 and lines 229-233: Plant phytol phosphate kinase can use any of the nucleotides (ATP, GTP, UTP or CTP) in their phosphorylation reaction. Authors should test the nucleotides other than CTP to see whether it is the only nucleotide used and not others.*

**Authors:** Thank you for your feedback. In spite that plant phytol kinases can use ATP, GTP, UTP, or CTP, they exhibit a preference for CTP (PMID: 16361393 and 16306049). Similarly, a limited number of other prenol kinases studied in nature also prefer CTP (PMID: 21395888, 8830051 and 9606952). Given these considerations, we decided to exclusively employ CTP in our research, since our primary goal was to identify catalytic activity and lipidic substrates, rather than delve into the specifics of phosphate donation or cofactor involvement. This decision was also influenced by the cost of assessing enzymatic activity, due the use of different radiolabeled precursors.

*8. Lines 249-257: Piggyback insertional mutagenesis screen by Zhang et al. showed this gene to be essential as it resisted being mutagenized. Since this is in contrast to the more robust findings reported here by the authors, it might be worthwhile pointing this out. The failure of Piggyback mutagenesis for this gene may be due to its small size. This addressed in Discussion (Lines 363-365) but might be important to raise in the Results section.*

**Authors:** Thank you for your suggestion. We also recognize the importance of addressing this phenomenon in the results section, and we have indeed done so (lines 446-451). See below:

“Previous studies employing piggyback mutagenesis indicated that the gene is essential in *P. falciparum* (Zhang et al, 2018), whereas studies in rodent malaria parasites predicted it to be dispensable (Bushell et al, 2017). Interestingly, Δ-PolK parasites remained fully viable with no observable growth rate changes, in agreement with the observations made in rodent parasites. We suggest that the failure of Piggyback mutagenesis for *Pf*PolK may be due to its small size.”

*9. Lines 336-340: Sentences are repeated.*

**Authors:** We thank the reviewer for spotting this mistake. We corrected this.

*10. Lines 350-353; See comment #3 above. These statements are incorrect.*

**Authors:** We corrected this and the phrase has been excluded. The text now reads “…Remarkably, this is the first FOH/GGOH kinase enzyme discovered in a non-photosynthetic organism. As observed in the phylogenetic analysis, putative prenol kinases comprise a very divergent group of clades. This finding supports the idea that Plasmodium's PolK is more similar to unicellular algae proteins, consistent with the *endosymbiosis event that occurred in apicomplexan ancestors… ” (lines 434-437)*

***Reviewer 2:*** *In this study, authors identify a novel kinase, PfPolK, and investigate its role in the farnesol (FOH) and geranylgerniol (GGOH) salvage pathway in malaria parasites. The authors use bioinformatic approaches to nominate candidate prenol kinases in Plasmodium falciparum using A. thaliana and Synechocystis spp. as references. Authors then completed a phylogenetic analysis of their candidate kinase, PfPolK, and found that it is most similar to enzymes in unicellular algae. Using Alpha-Fold, authors generate a model to suggesting that the PfPolK has a potential prenol binding pocket that could accommodate both FOH and GGOH. The investigators express PfPolK in S. cerevisiae, and find that yeast lysates from yeast expressing PfPolK can catalyze prenol monophosphorylatation with both FOH and GGOH. Using a conditional knockout of PolK in P. falciparum, the investigators find that the knockout strain has no growth defect, indicating PolK is not an essential enzyme for asexual parasite replication. However, PolK KD parasites no longer are rescued from fosmidomycin treatment by exogenous FOH or GGOH. These findings indicate that PolK may be the kinase responsible for phosphorylating exogenously supplied FOH and GGOH, which can be used to rescue inhibition of isoprenoid metabolism. Finally, authors show that the level of [3H]-FOH incorporation into proteins, as a measure of prenylation, is markedly reduced in the delta-PolK strain, further supporting a role for PolK for incorporation of farnesol.*

*Overall, this study successfully identified and characterized an enzyme, PolK, that catalyzes phosphorylation of prenols and is required for incorporation of exogenous prenols in Plasmodium falciparum. While this work provides insights into isoprenoid metabolism in P. falciparum, the clinical importance of these findings may be more modest than suggested by the authors. While parasites clearly are capable of prenol alcohol uptake and incorporation into downstream metabolites, it is not clear that they have access to relevant concentrations of prenol alcohols during human infection.*

**Authors:** We thank this reviewer for the consideration of the manuscript and the valuable insights provided. We agree with this reviewer that the primary focus of our article lies in the identification of the PolK enzyme, its activity and the relationship with parasite usage of prenols *in vitro*. Nevertheless, given the limited knowledge surrounding the FOH/GGOH salvage pathway, we consider important to propose potential biological roles of this pathway in malaria parasites. Thus, we have incorporated all the recommended modifications suggested by the reviewer, clearly indicating when we discuss hypotheses and maintaining a more restrained tone throughout our work. Notably, we have also included new data reinforcing our hypothesis regarding the potential biomedical importance of PolK (Figure 7) (lines 367-406). Although we are already embarked on research involving PolK inhibitors, the creation of *P. berghei* PolK knockouts, and the quantification of prenols in the human host organism, these studies will take time. Hence, we believe it is important to communicate the discovery of PolK so that the scientific community can explore its potential role in *Plasmodium*, or investigate PfPolk homologous genes in other organisms. For example, it has already been demonstrated that *Toxoplasma gondii* relies on both isoprenoid biosynthesis and host intracellular isoprenoids (PMID number: 24146616), a phenomenon which limits the efficacy of isoprenoid inhibitors against this parasitic infection. Therefore, it may be important to other researchers explore PfPolK homologs in *T. gondii*, as a viable strategy to enhance antiparasitic drugs. This issue is now included in text (lines 509-514)

***Reviewer 3:*** *This work identifies an enzyme capable of scavenging prenols for the synthesis of isoprenoids in blood-stage malaria parasites. Prenols, particularly genanylgeraniol (GGOH), were known to significantly reduce the sensitivity of P. falciparum parasites to isoprenoid synthesis inhibitors such as fosmidomycin. This phenomenon should require the prenols to be phosphorylated by a prenol kinase followed by formation of prenol pyrophosphates by a second kinase. Crispim and colleagues identified a potential prenol kinase (PolK) and characterized the role of this transmembrane enzyme in blood-stage parasites. Expression in yeast confirmed this enzymatic activity. Deletion of PolK in P. falciparum showed that this enzyme is dispensable, however, the deletion line was more susceptible to fosmidomycin and it could no longer be rescued with prenols. These results demonstrate the enzymatic activity of PolK and show that PolK is required for prenol scavenging, but also show that prenol scavenging is not essential under parasite culture conditions.*

**Authors:** We appreciate the feedback provided by the reviewer and we agree that the primary focus of our work revolves around the discovery of the PolK enzyme.

***Part II – Major Issues***

***Reviewer 2:***

*1) The biological significance of the prenol kinase activity of PolK is not clear. The authors discuss the possibility of dietary acquired FOH/GGOH as a potential mechanism for salvage in malaria parasites as a rationale for studying this protein. However, while the phosphorylated metabolite FPP has been detected in human serum, there is no known dietary derived FOH in human serum, or GGOH. Note that the human Metabolome database has FOH as having been detected in human blood https://hmdb.ca/metabolites/HMDB0004305 However, the sole reference cited (PMID: 9324945) detected FPP in human blood through conversion to FOH. GGOH is not reported to be found in the human metabolome. It seems plausible that the prenol kinase activity of PolK is a moonlighting activity of this enzyme, which may typically have some alternative metabolic function in the parasite. The references provided that suggest a biologically meaningful intake of prenols (e.g., mitigation of statins in a cancer model) do not mitigate this concern, as, for example, PMID: 28710496 employed a murine cancer model in which the diet was supplemented by 0.14 mg/mL GGol, a highly unphysiological concentration. In addition, farnesol is readily oxidized by cytochrome P450 enzymes and glucuronidated, which means that intraerythroycytic P. falciparum is unlikely to encounter this molecule in its unmodified form (PMID: 15320866).*

**Authors:**

We would like to express our gratitude for the reviewer's comments, as they helped to improve the presentation of the hypotheses and the whole manuscript. We have modified the text with the aim of clearly stating when we are discussing hypotheses, maintaining a more modest tone throughout our work. We performed these modifications in all the text (including the abstract), but we also include new data supporting the potential biological relevance of this prenol salvaging mechanism (see: lines 44-47, 156-160, 462-495, 520-524 and Figure 7).

Nevertheless, while we agree with the reviewer that exogenous prenols may not be the primary cause of fosmidomycin therapy failure in clinical settings, they could potentially contribute to a reduction in drug efficacy. Therefore, exploring this pathway might offer opportunities to enhance fosmidomycin's potency and reduce required dosages. Notably, ongoing research (PMID: 35111242) also investigates prenol production and dietary uptake in the human body, and our own published work demonstrated that food extracts diminish fosmidomycin efficacy (PMID: 36531309). Regarding the reference cited by the reviewer (PMID: 9324945), we decided to perform similar studies (Figure 7). We detected GGOH for first time in human plasma (not FOH) and provided new evidence that this compound, besides other prenols, could limit the efficacy of fosmidomycin in humans. In summary, the limited knowledge in this area together with our new data, suggest that some hypotheses warrant further investigation. Additionally, our findings may hold significance for other diseases. For instance, *T. gondii* relies on isoprenoid biosynthesis and host intracellular isoprenoids (PMID: 24146616), which limits the effectiveness of isoprenoid inhibitors against this parasitic infection.

We also appreciate the reviewer's concern regarding the potential moonlighting activity of PfPolK. However, while it is challenging to rule out alternative metabolic functions in the discovery of new enzymes, we believe that our study solidly confirms PfPolK prenol-phosphorylation activity using well-established techniques. In addition, drug-rescue assays in knockout parasites and radiolabeling experiments provide robust evidence for PolK's essential role in utilizing exogenous prenols.

*2) Fig. 2 – Conclusions re: AlphaFold modeling are somewhat overstated without experimental validation. For example, the authors propose “a novel prenol binding pocket” (line 359-360). Especially since they have a functional assay in hand, targeted mutagenesis of binding site residues should be used to confirm this model.*

**Authors:** We agree with the reviewer's suggestion**.** AlphaFold modelling shows a clear conserved binding site for the dNTP, and a hydrophobic channel that we propose to be the prenol binding site. However, as we do not have experimental support, we have now modified all data extracted from the molecular modelling, to avoid making conclusive statements about the hydrophobic channel (see lines 229-241 and 439-443).

*3) Figure 4 – quantification and statistical analyses of IF findings shown in panel G should be provided, to support the claim that PolK does not fully co-localize with anti-FNR. A brightfield image would also improve clarity.*

**Authors:** Please, see the new data (Figure 4H) including quantification and statistical analyses. Nevertheless, it is important to highlight that parasites treated with clindamycin (a well-known apicoplast disrupter) can still be rescued by FOH and GGOH (see Fig. 5A). This strongly suggests that PfPolK and the FOH/GGOH salvage pathway are not exclusively located within the apicoplast. In line with our findings and actual hypotheses, deep learning-based signal prediction tools indicate that this enzyme is localized in the endoplasmic reticulum of malaria parasites (PMID: 35489069). Moreover, other studies suggest that FOH/GGOH kinases, including FOLK, are predominantly located in non-plastidic compartments, such as the endoplasmic reticulum (PMID: 21395888; 36557825; 9606952). Regarding the brightfield image, while we do not have this for the primary immunofluorescence in Figure 4, we have included brightfield images of other replicates in the supplementary material (Figure S5C). We hope the reviewers will understand that they offer additional context and complement our primary immunofluorescence data, thereby enhancing the overall comprehension of our major objective in this experiment: to demonstrate the non-exclusive colocalization of *Pf*PolK with the apicoplast, thereby supporting the results of the clindamycin (apicoplast disruptor) rescue assays. We are confident that this objective has been successfully achieved with the data provided

*4) Discussion Lines 384-402 – this discussion should be softened considerably in the absence of additional evidence for prenol salvage occurring in Plasmodium spp.*

**Authors:** Again, we thank the reviewer’s comment. We have now made modifications to the text as suggested by the reviewer, maintaining a more measured tone throughout our work, while retaining some hypotheses, which can be precisely identified as such and supported by new experimental data (Figure 7). We performed these modifications not only in Discussion (lines 462-495) but also across the entire manuscript, including the Abstract (lines 44-47), Introduction (lines 156-160) and Conclusions (lines 520-524).

*5) For all experiments with the PolK knockdown line, methods should clarify duration of culture in the presence/absence of rapamycin.*

**Authors:** Thank you for the suggestion. We have included a description that clarifies the duration of culture in the presence and absence of rapamycin in the legends of figures 4 to 6, as well as in the methodology section (sections 5.7, 5.8, 5.9).

***Reviewer 3:***

*1) Given the dispensability of PolK and prenol scavenging it is not clear whether PolK is a worthwhile enzyme to target with inhibitors – even in the presence of isoprenoid synthesis inhibitors. Fosmidomycin does work against blood stage parasites in vivo and it is not known whether parasites in red blood cells have sufficient access to serum prenols to significantly affect fosmidomycin activity. One insight into this question could come from using different amounts of human serum (and possibly leukocytes) in the culture system and measure shifts in fosmidomycin susceptibility. Another insight would come from a titration curve showing how much GGOH is required to significantly rescue parasites treated with a concentration of fosmidomycin that can be achieved clinically (10uM?). Without information of this kind, it is hard to assess the possibility that PolK would be of interest to target pharmacologically.*

**Authors:** We have now made significant modifications through the text to maintain a more modest tone in our conclusions, clearly delineating when we discuss proposed hypotheses (lines 462-495, 44-47, 156-160, 520-524). In addition, we carried out several experiments suggested by the reviewer (Figure 7 and lines 367-406). We analysed AlbuMAX I lipid rich media supplement and human plasma by GC-MS, detecting for first time GGOH (not FOH). We also performed growth assays and compared fosmidomycin efficacy using whole or delipidated human plasma in culture. We agree that our study neither demonstrates nor aims to demonstrate that exogenous prenols are the cause of fosmidomycin therapy failure in **clinical settings** but to understand how prenols metabolization occurs in malaria parasites. However, our new results suggest that prenols could still play a relevant role, warranting further investigation. In addition, it is important to note that the concentration of prenols in blood, and the physiology of their absorption/excretion in humans remain largely unknown. Further research is needed to assess if the FOH/GGOH salvage pathway is used by the parasite to scavenge host prenols during infections, and to determine if this phenomenon might be connected to the limited efficacy of fosmidomycin. To address this, we have initiated research involving PolK inhibitors, the creation of *P. berghei* PolK (murine malaria parasites) knockouts, and the quantification of prenols within the human host organism. It's worth noting that these studies will span over several years.

In summary, our article certainly does not demonstrate a cause for fosmidomycin clinical failure, but it provides sufficient data to warrant further study into the biomedical importance of the FOH/GGOH salvage pathway.

*2) A related point connects to the discussion on fosmidomycin sensitivity in the PolK deletion line and the suggestion that prenols in the culture medium (from bovine albumin) may be responsible. They should address the question of bovine albumin being the source by using delipidated albumin reconstituted with oleate and palmitate. As mentioned above, careful attention should be paid to leukocytes. Although the investigators probably remove the buffy coat from the blood they use in culture, they should use a leukodepletion filter to make sure low levels of remaining leukocytes are also removed.*

**Authors:** We have carried out similar experiments to those suggested by the reviewer, using human plasma with leukocytes removed (Figure 7). Our results are compatible with the discussed hypothesis about the incorporation of host prenols. However, we really believe that our study just lays the groundwork for future investigations about the biomedical importance of the prenol salvage pathway. These investigations are complex and will require to study animal physiology and the kinetics of prenols metabolism in-depth.

*3) The localization of PolK should be further addressed experimentally. The current images are not clear and are not accompanied by quantitative co-localization with apicoplast and ER markers. The discussion of PolK localization at lines 371+2 should also point out that the Boucher used a BioID probe trafficked through the ER, making it potentially challenging to distinguish between ER proteins and apicoplast proteins.*

**Authors:** Thank you for your advice. We have now quantified and conducted statistical analyses of the IF findings, as suggested (see Figure 4H). In addition, we want to stress that more than precisely determining PfPolK location, our aim was to evaluate the independence of the apicoplast. For this, the IF analysis serves as a complementary approach, since clindamycin-treated parasites (an apicoplast disruptor) are rescued by FOH and GGOH. Hence, this observation suggests that the FOH/GGOH salvage pathway is not solely reliant on the presence of the apicoplast. In line with our findings and hypotheses, deep learning-based signal prediction tools indicate that *Pf*PolK is localized in the endoplasmic reticulum of malaria parasites (PMID: 35489069). Moreover, other studies suggest that FOH/GGOH kinases, including FOLK, are predominantly located in non-plastidic compartments, such as the endoplasmic reticulum (PMID: 21395888; 36557825; 9606952).

**Part III – Minor Issues**

***Reviewer 2:***

*1. Figure 2 – the overall conclusions of this* *phylogenetic analysis are relatively modest, especially since many of these enzymes have not been confirmed as prenyl kinases. This* *figure could be moved to the supplemental data and conclusions softened.*

**Authors:** As suggested by the reviewer, we have enhanced the phylogenetic analysis and made text modifications to soften our conclusions. However, we would like to keep the phylogenetic analysis, as it was instrumental in identifying the PolK candidate in malaria parasites. We believe that this data could be valuable for future studies involving prenol kinases in other organisms.

*2. Figure 3 – it is interesting that this kinase uses CTP. Did the investigators try additional phosphodonor substrates, including other nucleotides? In addition, the position of standards is indicated but should be explicitly shown, ideally on the same TLC plate as the enzyme assays.*

**Authors:** A limited number of other prenol kinases studied in nature also tend to favor CTP as their choice of nucleotide. Considering this, we made a deliberate choice to exclusively employ CTP in our research. It's important to emphasize that our primary focus was to identify the catalytic activity and the lipidic substrate, rather than delve into the specifics of phosphorous donation or the involvement of cofactors. It's essential to acknowledge that this decision was also influenced by the costliness of assessing enzymatic activity, particularly because it involves the use of expensive radiolabeled precursors. About the standard position, we conduct chromatography of non-radioactively labelled standards in parallel with the samples. Once the chromatographic analysis is complete, we observe the standards migration using iodine vapor and/or UV light. We mark the results with a pencil because iodine tends to evaporate over time, and the En3Hance spray eliminates the fluorescence. This way we can indicate the retention of the different standards. However, consequently, after autoradiography we are unable to display images of the specific standards used in each experiment. To address the concerns raised by the reviewer, we have included a photograph in the supplemental data showing the three standards (prenol, prenyl-P, and prenyl-PP) visualized using iodine vapor (Figure S7).

*3. Figure 4 –* *Panel F would benefit from reprobing with a control antibody to confirm equal transfer efficiency.*

**Authors:** We have now included in supplemental data (figure S5B) a new Western blot analysis showing similar results and including a Ponceau S staining, as loading control. Lastly, the source of the anti-HA antibody has now been addressed in Materials and Methods (section 5.11) and we have adjusted the font sizes.

*4. Figure 6 – use of prenyl transferase inhibitors to interrupt protein prenylation is recommended as a control to confirm that the incorporation is specific.*

**Authors:** Recently, we published several SDS-PAGE gels of parasitic proteins radiolabelled with prenols, and we also conducted proteomic studies of the radiolabelled bands to assess the presence of proteins predicted to be prenylated (PMID: 36531309). We observed a very specific incorporation of prenols into 2-3 specific protein clusters containing the prenylated proteins of parasites (see Figure 4, at Verdaguer et al. 2022, PMID: 36531309; please see a snippet of the figure below). As it can be observed in the image, tritiated farnesol and geranylgeraniol are incorporated into proteins forming clear clusters of distinct molecular weights. Given these findings, we decided to shift our focus towards assessing general protein synthesis control using radiolabelled leucine.

**SDS-PAGE analysis of [^3^H] FOH and [^3^H] GGOH-labeled proteins in *P. falciparum*.** Synchronous cultures at the ring stage were labeled with either 0.75 μCi/ml of [^3^H] FOH or [^3^H] GGOH. After 12–16 hours, parasites at the trophozoite/schizont stages were treated with saponin and then solubilized in SDS sample buffer for SDS-PAGE analysis

*5. Even modest reductions in protein prenylation in P. falciparum have been shown to be important for surviving heat shock (PMID: 34182772). Although there is no growth defect under normal asexual replication in the PolK strain, have the authors assessed survival of this strain under heat or cold shock?*

**Authors:** Thank you for your advice. We were unaware that reductions in protein prenylation in *P. falciparum* are crucial for surviving heat shock, so we did not assess the survival of this strain under heat or cold shock conditions. However, as the reviewer pointed out, there is no growth defect observed during normal asexual replication in the PolK strain. This observation is consistent with the absence of significant differences in protein prenylation, showing that the FOH/GGOH salvage pathway is not essential.

***Reviewer 3****:*

*-Fig 3. Standards are not shown for polyprenyl-P and polyprenyl-PP. Densitometry of the bands in the 3 replicate autoradiographs should allow the authors to provide statistics in addition to showing the images for one of the replicates.*

**Authors:** Normally, we conduct chromatography of non-radioactively labelled standards in parallel with the samples. Once the chromatographic analysis is complete, we observe the standards using iodine vapor and UV light. We mark the results with a pencil because iodine tends to evaporate over time, and the En3Hance spray eliminates the fluorescence. Consequently, after autoradiography, we are unable to display images of the specific standards used in each experiment. This way we can indicate the retention of the different standards. To address the concerns raised by the reviewer, we have included a new supplementary figure (Figure S7) showing the three standards (prenol, prenyl-P, and prenyl-PP) visualized using iodine vapor.

Furthermore, it should be noted that we cite some articles employing the same chromatographic system for the detection of the same compounds. As observed in the literature (PMID: 16361393; 21395888; 8830051; 9606952; 16306049), enzymatic activities of PolK enzymes and other lipid kinases assayed through radio-TLC, are commonly interpreted based on the visualization of products. Therefore, we believe that it is not necessary to analyze the results using densitometry. It's important to note that the autoradiographies are clear and precise (i.e., the prenyl-P band is either present or absent, without ambiguities in the spots).

*-Figure 4A. This genotyping strategy does not distinguish between integrants and the transfection plasmid. The insertion should be verified with primer pairs that flank each homology arm to demonstrate integration into the targeted locus.*

**Authors:** In response to the critique on Figure 4A, it should be noted that the gDNA from the transfected parasite clones was subjected to two separate PCR analyses. One is previous to the confirmation of the *Pf*PolK gene excision, and specifically differentes wild-type *Pf*PolK and the recodonized *Pf*PolK gene (Figure 4C). The second PCR specifically shows the disruption of recodonized PfPolK (Figure 4D). Thus, Figure 4C shows complete integration into the locus and absence of the wild-type *Pf*PolK gene. We believe that the absence of amplification of the wild-type *Pf*PolK gene in the transfected parasites confirms its replacement by the recodonized version. Finally, it should be noted that the complete edition of the gene in parasites is also demonstrated by the appearance of phenotypes different from parental parasites, the detection of HA-fused protein by Western blot, and immunofluorescence.

*-Figure 4e. The figure legend describes ‘SD of at least three experiments’, but the plots do not seem to have any error bars.*

**Authors:** We appreciate the reviewer's observation. The sentence in question did not correspond to the graph and has been removed. Please, see that the independent analyses of three different mutant clones, with comparable results, could potentially serve as a viable alternative to biological replicates

*-Fig S5.* *In the Western blot, lower band.*

**Authors:** Sorry, we didn’t understand the reviewer’s comment. Is it possible that the reviewer wants some sort of explanation to the intense low molecular weight band that appears in lane 2, but not in lane 1? If so, we think it is an artifact of the WB. Please notice that this band does not appear in the additional Western blot image now included in the Supplemental Figure S5B.

*Line 177. The word ‘similarity’ in pairwise sequence alignments requires definition. The authors may have meant ‘identity’ here.*

**Authors:** We actually meant "identity" and changed the word in lines 189 and 190. We apologise for this mistake and thank the reviewer for pointing it out. We calculated the identity as a percentage considering identical amino acids between sequences, data from UniProt. For the sake of clarity, we have briefly defined it in the text, as recommended by the reviewer (see lines: 188-191).

*-Lin 187. Acquisition of PolK through the algal endosymbiont would be most consistent with apicoplast localization of PolK, but this does not seem to be the case (or at least not exclusive apicoplast localization).*

**Authors:** We believe that apicoplast localization of PolK may not necessarily suggest its acquisition through the algal endosymbiont. It is known that several endosymbiont genes were transferred to the parasitic nucleus, and some of the products of these genes were relocated to other cellular compartments (PMID: 18353992). In line with our findings and actual hypotheses, deep learning-based signal prediction tools indicate that this enzyme is localized in the endoplasmic reticulum of malaria parasites (PMID: 35489069). Moreover, other studies suggest that FOH/GGOH kinases, including FOLK, are predominantly located in non-plastidic compartments, such as the endoplasmic reticulum (PMID: 21395888; 36557825; 9606952). Additionally, as previously mentioned, the fact that parasites treated with clindamycin (an apicoplast disruptor) can still be rescued by FOH and GGOH strongly indicates that the enzymes of the FOH/GGOH salvage pathway are, at least, not exclusively localized within the apicoplast.

*-Line 198. Replace ‘of the Phe43 and Ile71 to fit the later.’ With ‘**of Phe43 and Ile71 to fit the latter.’*

**Authors:** Thank you, we did this correction. (see line 237-238)

*-Line 238. Autoradiographies ->* *autoradiographs*

**Authors:** Thank you, we did this correction as suggested by reviewer. (see line 260)

*-Line 252. ‘In contrast’ -> ‘By contrast’*

**Authors:** We did this correction as suggested by reviewer. (see line 274)

*-Line 359. Molecular modeling is not conclusive and statements, such as those in line 359 should be moderated (‘the nucleotide ring appears to be stabilized’, ‘The model also suggested a novel’)*

**Authors:** We completely agree with the reviewer's suggestion and we have modified all data extracted from the molecular modeling to avoid making conclusive statements. Changes are now included in the Results (lines 230-239), Discussion (lines 439-443) and Materials and Methods (lines 634-643) sections.

*-Line 401. Change ‘showed to be slightly more susceptible to fosmidomycin.’ To* *‘displayed greater sensitivity to fosmidomycin.’*

**Authors:** We changed this exactly as suggested by the reviewer (see line 462).

*-Line 407. Change ‘malaria’ to ‘malaria parasites'*

**Authors:** Thank you, we changed this in line 496.

*-Line 449. The methods state that polyprenyl phosphates were prepared by acid treatment of commercial pyrophosphates. The issue here is that the polyprenyl phosphates are then used as a standard for the enzymatic assays, but there wasn’t clear evidence presented on the synthesis or purity of these polyprenyl phosphate standards.*

**Authors:** To the best of our knowledge, commercial polyprenyl phosphates were not commercially available until recently. Although currently accessible through Sigma-Aldrich, these reagents are very expensive and the delivery time to our lab takes several months. Therefore, we usually prepare polyprenyl phosphates by subjecting commercial pure pyrophosphates to acid treatment (the Sigma references of the commercial polyprenyl-PP used are now indicated in text, in line 538). As mentioned in the text, this process is already documented in the literature (PMID: 8830051) and, from a chemical standpoint, it is difficult to create artifacts with chromatographic retention compatible with polyprenyl phosphates. Again, it should be noted that we cite an article in which this method is used (PMID: 8830051), and several other that employ the same chromatographic system and procedures for the detection of the same compounds (PMID: 16361393; 21395888; 8830051; 9606952; 16306049). To address the concerns raised by the reviewer, we have now included an image in the supplemental data Figure S7 showing the three standards (prenol, prenyl-P, and prenyl-PP) visualized using iodine vapor and UV light.

*-Line 486. ‘it was calculated the dose-response curve’ -> ‘the dose-response curve was calculated’*

**Authors:** We changed this exactly as suggested by the reviewer (see line 575).

*-Line 495 ‘data was’ -> ‘data were’*

**Authors:** Thank you, we didn't notice this mistake. We have corrected it (see line 581).

*-The transfection plasmid sequence (particularly the recoded part) should be provided as supplemental material (or a database submission).*

**Authors:** Yes, we agree with the reviewer and the transfection plasmid sequence is now provided as supplemental material (Figure S6).
