# Supplementary material for: Beyond the MEP Pathway: A novel kinase required for prenol utilization by malaria parasites
Source: PLoS Pathog. 2024 Jan 26;20(1):e1011557. doi: 10.1371/journal.ppat.1011557 (PMC10849223; doi:10.1371/journal.ppat.1011557)
Supplement: Attachment — Submitted filename: Responses to reviewers 12.docx [file ppat.1011557.s009.docx]

**Responses to reviewers and editors**

***Editor comments:***

*Dear Dr. Katzin,*

*Thank you very much for submitting your manuscript "Beyond the MEP Pathway: a novel kinase required for prenol utilization by malaria parasites" for consideration at PLOS Pathogens. As with all papers reviewed by the journal, your manuscript was reviewed by members of the editorial board and by several independent reviewers. The reviewers appreciated the attention to an important topic. Based on the reviews, we are likely to accept this manuscript for publication, providing that you modify the manuscript according to the review recommendations.*

*All three reviewers were quite complementary regarding the extent of the revisions and your attention to their suggestions. They each made additional comments that can be addressed through changes to the text. Reviewer 3 also noted that the diagnostic PCRs regarding the genetic modications should be more accurately performed. This is a relatively simple experiment that will require little effort and should be done. Reviewer 2 suggested experiments involving delipidation of human serum and additional validation of TLC standards. These are valuable suggestions, however given that we do not want to delay publication of the manuscript, addition of these experiments will be left to the authors' discretion.*

**Authors:** We thank editors and reviewers for helping in the revision process of our manuscript. By attending the reviewers’ critiques and comments, we believe the paper has been much strengthened. In this second revision phase we have further addressed the suggested points and have carried out the key recommended experiment. We really believe that we are providing a modified manuscript according to the review recommendations. It's important to comment that we wish to make the revision process public, as we believe our responses to the reviewers could be beneficial to other authors. Here is a summary of the new changes in the manuscript and issues discussed with reviewers as outlined in this document:

-All the reviewers’ additional comments have been addressed through changes to the text. Please, see our responses to reviews below.

-As reviewer 3 suggested, the diagnostic PCRs regarding the genetic modifications has been performed and included in the article. Please find the corresponding modifications in the Methods (section 5.9) and Results (section 2.3 plus Figure 4 and its respective legend).

-As the editors pointed out, reviewer 2 suggested experiments involving validation of TLC standards. As previously commented, Sigma-Aldrich commercial polyprenyl phosphates, are very expensive and the delivery time to our lab in Brazil takes several months. Therefore, we decided to prepare polyprenyl phosphates by subjecting commercial pure pyrophosphates to acid treatment. We would like to emphasize that this process is already documented in the literature (PMID: 8830051) and, from a chemical standpoint, it is improbable to create artifacts with chromatographic retention compatible with polyprenyl phosphates. We further cite an article in which this method is used (PMID: 8830051), and several other that employ the same chromatographic system and procedures for the detection of the same compounds (PMID: 16361393; 21395888; 8830051; 9606952; 16306049). Nevertheless, we tried to address the concerns raised by the reviewer by including more explanations (see reviewer response below) and by adding supplemental data, Figure S7. It should be noted that our approaches are identical to or even slightly improved compared to those of all the other authors in the literature who studied prenol kinases.

- As the editors pointed out, reviewer 2 suggested experiments involving the delipidation of human plasma. We believe that these newly added experiments confirm that geranylgeraniol is present in plasma. However, we do not make a conclusive assertion that its sole presence in plasma affects fosmidomycin's efficacy *in vitro* or *in vivo*. We conclude that some solvent-removable lipids of human plasma affect fosmidomycin's efficacy and that its 'rescue' effects are dependent on PolK. We support this assertion based on the differential effects observed in the delipidation of human plasma between knockout and wild-type parasites. We further discuss that our assertions are correct, even though there is the possibility that prenols were not completely removed or might be present in red blood cells, as we deliberately discussed it in the manuscript. It should be noted that we employed very aggressive delipidation processes which are well-established in the literature and used by several authors; thus, we don’t think we should question their efficacy in removing lipids. Please, see a more detailed discussion about this topic in our responses to reviewer 2.

-We found a mistake in one author’s affiliation. We changed “Barcelona Centre for International Health Research, Hospital Clínic-Universitat de Barcelona, Barcelona, Spain” to “Barcelona Institute for Global Health (ISGlobal), Hospital Clínic-Universitat de Barcelona, Barcelona, Spain”.

We hope that the revised manuscript will now be acceptable for PloS Pathogens and we look forward to hearing from you.

**(The text lines indicated in the following responses to reviewers, correspond to the version of manuscript without changes indicated, file: Crispim et al 19)**

***Part I - Summary***

***Reviewer #1:*** *Crispim et al. have extensively revised their manuscript in response to its initial review. The manuscript now uses more moderate tone in stating the significance of the findings. In addition, the authors describe new experiments using human plasma that further bolster their suggestion that PfPolK is likely to play phenol salvage role in human infection. Overall, the manuscript is much improved. There may be some minor corrections that may be needed (e.g. Neisseria is NOT an apicomplexan, see line 210.)*

**Authors:** We thank reviewer 1 and we are grateful to have addressed their comments/concerns. Thank you for pointing out the mistake regarding Neisseria; we have corrected this (see lines 209- 210).

***Reviewer #2:*** *The authors are applauded in their comprehensive response to my queries and to those of the other reviewers. In particular, the new data is appreciated and helpful. However, a number of queries remain, and I still find many statements to be overly confident or broadly applied, inconsistent with the data presented.*

**Authors:** We thank reviewer 2. We are grateful that the reviewer appreciated the new experiments. Please note that we have further addressed all the suggested points and have carried out an additional experiment, as suggested by the reviewer. We hope that the revised manuscript will now be acceptable for publication.

***Reviewer #3:*** *The authors did a good job overall of responding to the reviews including providing new data that strengthen the paper. Below are some points to improve the paper with only one substantive point – as described below, the genotyping PCRs are not adequate. The phenotypes described in the paper make it extremely likely that the conditional KO line has the wrong genotype, but it is not a good idea to infer genotypes from phenotypes. It is best practice to fully establish the genotype and use that information to provide explanations for observed phenotypes.*

**Authors:** We thank reviewer 2 for its comments and further pointing out aspects to improve. As suggested by the reviewers, we have addressed the genotyping PCR. We hope that the revised manuscript will now be acceptable to the reviewer.

***Part II – Major Issues: Key Experiments Required for Acceptance***

***Reviewer #1:*** *(No Response)*

***Reviewer #2:*** *Experimental concerns:*

*1. New Figure 7 answers my query as to whether GGOH is found in human serum. However, the authors should confirm that GGOH is depleted by delipidation, since delipidation appears to increase fosmidomycin sensitivity, which they attribute to loss of GGOH.*

**Authors:** We appreciate the reviewer's comment. We would like to note that these newly added experiments confirm that geranylgeraniol is present in plasma. However, we do not make a conclusive assertion that its sole presence affects fosmidomycin's efficacy in vitro. We conclude that some solvent-removable lipid(s) of human plasma affect fosmidomycin's efficacy and that its 'rescue' effects are dependent on PolK. These results strongly support that this assertion is true based on the differential effects observed between knockout and wild-type parasites cultivated with whole/delipidated plasma. We further discuss in text that our assertions are correct, even though there is the possibility that prenols were not completely removed or might be present in red blood cells. It should be noted that we employed very aggressive delipidation processes which are well-established in the literature and used by several authors; thus, we don’t think we should question their efficacy in removing lipids.

*2. Appreciate the new inclusion of Supplemental Figure S7 to indicate position of standards. However, there can be run to run variability in standards position – it would be better to show these standards on the same TLC plate as used for the enzymatic data shown in Figure 3 (e.g., appreciating that the iodine marks fade, a photo of the same plate including controls such as in the Figure S7). Including the controls as shown confirms the relative positions of the prenol, prenyl-P, and prenyl-PP but does not confirm whether the new bands are in fact the prenyl-P vs. the prenol-PP, since no prenyl-PP is present for comparison. I remain concerns (as per reviewer 3’s query) that the identity and purity of these standards is not certain.*

**Authors:** We appreciate the reviewer comment, but we still feel concerned about not having addressed their comments fully. We cannot provide the figure which the reviewer requested because, in these kind of experiments, we observe several substances in samples (consistent with the numerous metabolites present in a yeast whole extracts), but we do not observe any iodine marks appearing or disappearing due to the low concentration of the radiolabeled enzymatic substrate (much hydrophobic) which can be added to the reaction. The inclusion of small amounts of detergents is performed to enhance substrate/enzyme solubility. However, as other authors have described, this strongly inhibits PolK activity (PMID: 16361393; 21395888; 8830051; 9606952; 16306049). In our case, following the experiences of other authors, we haven't tested the possibility of adding great amounts of detergents to increase substrate solubilization. Thus, the transformation of the radiolabeled substrate only becomes evident after radiographic exposure. That's why we (and all the authors who studied enzymes similar to PolK) employ radiolabeled substrates (PMID: 16361393; 21395888; 8830051; 9606952; 16306049). It's important to comment that we still possess the original TLC plates of all enzymatic activities, but the iodine marks are no longer visible. As previously commented, once the chromatographic analysis is complete, we observe the standards' migration using iodine vapor and/or UV light. We mark the results with a pencil because iodine tends to evaporate over time, and the En3Hance spray eliminates the fluorescence. Furthermore, it should be noted that this enzymatic assay is expensive and requires weeks of exposure to radiographic films.

Regarding the concerns about compounds identity: As previously commented, Sigma-Aldrich commercial polyprenyl phosphates, are very expensive and the delivery time to our lab in Brazil takes several months. Therefore, we decided to prepare polyprenyl phosphates by subjecting pure commercial pyrophosphates to acid treatment. We would like to emphasize that this process is already documented in the literature (PMID: 8830051) and, from a chemical standpoint, it is unexpected to create artifacts with chromatographic retention compatible with polyprenyl phosphates. We further cite an article in which this method is used (PMID: 8830051), and several other that employ the same chromatographic system and procedures for the detection of the same compounds, with similar retention factors (PMID: 16361393; 21395888; 8830051; 9606952; 16306049). It should also be noted that the newly-formed second band in enzymatic assays appears only when an unlabeled phosphate donor is added, which demonstrates the phosphorylation of the radiolabeled substrate. To our knowledge, no molecules are known to be formed by the condensation of CTP plus a prenol, except prenyl phosphates. Furthermore, no enzyme known in nature is capable of directly producing prenyl pyrophosphates through double phosphorylation from prenols. Nevertheless, we tried to address the concerns raised by the reviewer by including the image in the supplemental data, Figure S7. Considering all this, we believe that our approaches are similar to or even slightly improved compared to classic methods employed by all the articles in the literature who studied prenol kinases.

*Major concerns with data interpretation/discussion:*

*3. Discussion paragraph lines 439-443 still overstates the conclusions from the Alpha Fold structural model. Would suggest that the authors make it clear that this is a computational model that awaits experimental validation. In addition, 3. with protein in hand they should be able to determine catalytic efficiency for GGOH vs. FOH substrates directly if this is of interest, so the hypothesizing based on modeled binding energies should be removed.*

**Authors:** We agree with the reviewer's comment. As suggested by the reviewer, we have included in the text that “…Despite these remarks, it should be noted that all these computational predictions need further experimental validation” (literally; see lines 452 and 453). We have also softened sentences that hypothesize based on modeled binding energies and made it clear again that these are just computational predictions awaiting experimental validation (see lines 441-453). However, we still maintain the figure because we think that explaining that the model shows a putative prenol binding pocket could be of importance for other researchers.

*4. Line 472 – it is not clear from the data provided that this pathway is used in vivo for scavenging. It is still possible that the essentiality of this pathway is limited to the non-physiologic laboratory scenario of isoprenoid inhibition in vitro. The de Wolf paper should not be cited as “dietary sources" of GGol – these animals were forced to ingest high quantities of pure GGol. As written, the authors imply that there are foods that could contain sufficient GGol to complement deficient or inhibited parasites.*

**Authors:** We agree with the reviewer's comment. We also believe that it's still possible that the essentiality of this pathway is limited to the non-physiological laboratory scenario. Please note that we have further softened our text indicated that the geranylgeraniol-rescue phenomenon observed by de Wolf et al. requires further research to confirm whether it may occur naturally (lines 506-515). We have also indicated that, at this time, no evidence has been presented to suggest the existence of dietary sources of geranylgeraniol capable of rescuing parasites from MEP inhibitors. Therefore, this is an aspect that should be further studied in the future. In our previous corrections, we already made clear when we hypothesized about the occurrence of prenol-rescue *in vivo*. Our study neither demonstrates, nor aims to demonstrate, that exogenous prenols are the cause of fosmidomycin therapy failure **in clinical settings**, but to understand how prenols metabolization via PolK occurs in malaria parasites *in vitro*. Our new results employing human plasma only open new avenues for research, such as the determination of prenol concentration in blood, and the physiology of their production/absorption/excretion in humans. Further research is needed to assess if the FOH/GGOH salvage pathway is used by the parasite to scavenge host prenols (either endogenously produced or coming from dietary sources) during infections, and to determine if this phenomenon might be connected to the limited clinical efficacy of fosmidomycin.

**(The following reviewer comment -num. 5- has been divided by the authors in order to better respond point-by-point to all the issues raised)**

*5. Discussion paragraph 496-514 still substantially overstates the role of dietary geranylgeraniol. The “cancer therapy” referenced is an HMG CoA reductase inhibitor that specifically inhibits isoprenoid production – the de Wolf study indicates that massive amounts of dietary GGol (0.14 mg/mL body weight) abrogated the antitumor effect in an HMG CoA reductase expressing ovarian cancer model - of course, no data is provided that this in vivo effect is on target. That is hardly generalizable as “implications for cancer therapy” since statins are not used clinically to treat cancer and that amount of dietary GGol is entirely unphysiologic…*

**Authors:** We agree with the reviewer's comment and thus, we modified the referred paragraph. We have indicated that the geranylgeraniol-rescue phenomenon in animal models described by de Wolf et al. was observed after unphysiologic supplementation and thus, it requires further research to confirm whether it may occur naturally (lines 506-515). See below:

*“…However, it should be noted that de Wolf et al. performed supplementation assays, which may lead to unphysiological concentrations of GGOH in mice, and thus, further research is required to confirm whether this occurs naturally”*

Please note that in the manuscript we have not indicated or discussed any evidence for the existence of dietary sources of geranylgeraniol capable of rescuing parasites from MEP inhibitors. Therefore, this is an aspect that should be further studied in the future and so we comment in text. At this point, we would like to clarify that the attractiveness of PolK as a target to enhance fosmidomycin efficacy is not solely discussed based on the **hypothesis** that geranylgeraniol in the blood may naturally rescue treated parasites. We also **hypothesize** that PolK may be involved in the endogenous recycling of prenols or in the metabolic regulation of isoprenoid metabolism by the parasite. Regardless of the biological function of this pathway, the fact that PolK KO parasites are more sensitive to fosmidomycin requires us to offer readers with plausible hypotheses that provide justification.

*…This is also a preclinical animal model, not a human “clinical trial” as stated in line 501….*

**Authors:** Thanks for pointing out this issue. This phrase has been replaced (lines 510-513).

*…The additional 2 references--Prior and Healy--are review articles and do not provide additional experimental evidence to this effect, and should be removed from the reference list in my opinion. As cited, the reader is led to believe that 3 different clinical studies found that dietary GGol impacts clinical cancer treatment, which I find misleading). The authors are additionally directed to an increasing body of literature that suggests a lack of anticancer efficacy of statins, despite the prominence of Ras mutations as oncogenic drivers (e.g., PMID: 31591592).*

**Authors:** We added Prior and Healy references to better discuss the metabolic implications of GGOH presence in food. Nevertheless, we agree with the reviewer that these references are not necessary, and thus, we have removed them. Additionally, we have better indicated that the geranylgeraniol-rescue phenomenon in animal models described by de Wolf et al. was observed after supplementation that may lead to unphysiologic GGOH concentrations (lines 506-515). Finally, we modified text to further made it clearer that statins are currently studied for cancer treatment, but only “in an experimental context” and that are not routinely employed for cancer therapy in human clinics (lines 506-515).

***Reviewer #3:*** *Genotyping PCRs in panel C currently show the absence of the WT gene (with control) and presence of the transgene (with control), but don’t demonstrate that the transgene was inserted in the targeted locus. Loss of the WT amplicon in the transgenic lines is highly suggestive of change at the WT locus, but not sufficient proof (negative data). The PCRs in panel C should have been designed to show insertion into the locus using primers that flank the homology arms. The diCre excision experiments in panel D aren’t the best evidence that the transgene is in the targeted locus, because the primers are in the homology arms (according to the description in the methods). Similar PCR amplicons (including diCre excision) could be obtained from retained transfection plasmid. The conditional KO parasite lines are almost certainly correct, but the genotyping PCRs shown in the paper are not the right way to prove it.*

**Authors:** We agree with the reviewer's comment. Thus, the diagnostic PCRs regarding the genetic modifications has been performed and included in the article. Please find the corresponding modifications in the Methods (section 5.9) and Results (section 2.3, plus Figure 4 and its respective legend).

***Part III – Minor Issues: Editorial and Data Presentation Modifications***

***Reviewer #1:*** *(No Response)*

***Reviewer #2:*** *1. Line 393 – Figure legend for Figure 7 is incorrect. No explanation of A is provided (looks like EIC for GGOH?).*

**Authors:** Thanks for pointing out this issue. We corrected the legend for Figure 7 and added explanations for “A”.

*2. Thank you for the image quantification in Figure 4H. I haven’t seen co-localization presented as “percentage overlap” before in other publications. Pearson’s correlation is more commonly used. Can the authors provide justification?*

**Authors:** Thanks for pointing out this issue. The Percentage of Overlap is already used by other authors (PMID: 24696464), and it was first described by Manders *et al*, 1993 (PMID: 33930978). We modified the methodological section to better explain how this analysis was performed and we also referenced the previously cited authors (Lines 735-736).

*3. A number of minor grammatical mistakes are noted in the discussion paragraph beginning on line 462. Additional copy-editing is recommended.*

**Authors:** Thanks for pointing out this issue. We have reviewed and corrected the grammatical mistakes in the paragraph beginning on the previous line 462 (now it corresponds paragraph beginning on line 472).

***Reviewer #3:*** *In the response to the reviews, the authors suggest that apicoplast disruption experiments with clindamycin are further evidence that PolK exists outside the apicoplast. I don’t think this point is made in the paper, but it should be modified if it is. Recent studies show that apicoplast enzymes can be active in the vesicles that remain after apicoplast disruption.*

**Authors:** We appreciate the reviewer's comment. In the text, we discuss that PolK exists outside the apicoplast only based on IF assays. We also comment that prenol-rescue of clindamycin occurs in WT parasites (a fact also demonstrated by other authors) and not in KO parasites, indicating that the prenol salvage pathway is not inhibited by this antibiotic. Whether this pathway remains active after clindamycin treatment in apicoplast-derived vesicles or in other cellular locations is not discussed, as our data do not support any conclusions regarding this issue. In any case, it is evident that the prenol salvage pathway remains active independently of the presence of apicoplast as a whole organelle. Importantly, this fact differs from MEP pathway which is indirectly inhibited by clindamycin and cannot remain active in apicoplast-derived vesicles or any other cellular localizations.

*In the results and discussion text describing the PiggyBac mutagenesis screen results, be sure use “PiggyBac” rather than “Piggyback”.*

**Authors:** Thanks for pointing out this issue. We corrected this issue (lines 457 and 460).

*Line 209: “Alveolate group,also yielded” Add space before “also”*

**Authors:** This phrase was removed due previous reviewer suggestion (line 209).

*Line 232: Change “The presumably CTP binding pocket” to use the word “putative” or “predicted” instead of “presumably”.*

**Authors:** Thanks for pointing out this issue. We corrected this issue and now we employ the word “putative” (lines 232 and 241).

*Figure 5 legend: “was monitored by either flow cytometry.” Is the word “either” needed here? Or, was another technique like SYBR green staining also used?*

**Authors:** Thanks for highlighting this issue. Yes, in figure legend 5, the word "either" is unnecessary since the results were exclusively monitored by flow cytometry. We've made the correction (line 352).

*Line 442: change “presumibly” to “possible” or “predicted”*

**Authors:** Thanks for pointing out this issue. We corrected this issue (line 451).

*Line 492: Change “further studies should being carried” to “further studies should be carried”*

**Authors:** Thanks for pointing out this issue. We corrected this issue exactly as suggested by the reviewer (line 502).

*Line 778: Change “we conducted experiments verify the” to “we conducted experiments to verify the”*

**Authors:** Thanks. We corrected this issue exactly as suggested by the reviewer (line 797).
